# Three-Dimensional Visualization and Detection of the Pulmonary Venous–Left Atrium Connection Using Artificial Intelligence in Fetal Cardiac Ultrasound Screening

**DOI:** 10.3390/bioengineering13010100

**Published:** 2026-01-15

**Authors:** Reina Komatsu, Masaaki Komatsu, Katsuji Takeda, Naoaki Harada, Naoki Teraya, Shohei Wakisaka, Takashi Natsume, Tomonori Taniguchi, Rina Aoyama, Mayumi Kaneko, Kazuki Iwamoto, Ryu Matsuoka, Akihiko Sekizawa, Ryuji Hamamoto

**Affiliations:** 1 Department of Obstetrics and Gynecology, Showa Medical University School of Medicine, 1-5-8 Hatanodai, Shinagawa-ku, Tokyo 142-8666, Japan; ryu@med.showa-u.ac.jp (R.M.); 2 AI Medical Engineering Team, RIKEN Center for Advanced Intelligence Project, 1-4-1 Nihonbashi, Chuo-ku, Tokyo 103-0027, Japan; 3 Division of Medical AI Research and Development, National Cancer Center Research Institute, 5-1-1 Tsukiji, Chuo-ku, Tokyo 104-0045, Japan; 4 Department of NCC Cancer Science, Biomedical Science and Engineering Track, Graduate School of Medical and Dental Sciences, Institute of Science Tokyo, 1-5-45 Yushima, Bunkyo-ku, Tokyo 113-8510, Japan; 5 Digital Health Platform Development Office, Healthcare Business Unit, Fujitsu Japan Ltd., 1-5 Omiya-cho, Saiwai-ku, Kawasaki 212-0014, Japan; 6 Department of Obstetrics and Gynecology, Graduate School of Medicine, The University of Tokyo, 7-3-1 Hongo, Bunkyo-ku, Tokyo 113-8655, Japan; 7 Department of Obstetrics and Gynecology, Tohoku University Graduate School of Medicine, 2-1 Seiryomachi, Aoba-ku, Sendai 980-8574, Japan

**Keywords:** total anomalous pulmonary venous connection, fetal cardiac ultrasound screening, PV-LA connection, 3D segmentation

## Abstract

Total anomalous pulmonary venous connection (TAPVC) is one of the most severe congenital heart defects; however, prenatal diagnosis remains suboptimal. A normal fetal heart has a junction between the pulmonary venous (PV) and left atrium (LA). In contrast, no junctions are observed in patients with TAPVC. In the present study, we attempted to visualize and detect fetal PV-LA connections using artificial intelligence (AI) trained on the fetal cardiac ultrasound videos of 100 normal cases and six TAPVC cases. The PV-LA aggregate area was segmented using the following three-dimensional (3D) segmentation models: SegResNet, Swin UNETR, MedNeXt, and SegFormer3D. The Dice coefficient and 95% Hausdorff distance were used to evaluate segmentation performance. The mean values of the shortest PV-LA distance (PLD) and major axis angle (PLA) in each video were calculated. These methods demonstrated sufficient performance in visualizing and detecting the PV-LA connection. In terms of TAPVC screening performance, MedNeXt-PLD and SegResNet-PLA achieved mean area under the receiver operating characteristic curve values of 0.844 and 0.840, respectively. Overall, this study shows that our approach can support unskilled examiners in capturing the PV-LA connection and has the potential to improve the prenatal detection rate of TAPVC.

## 1. Introduction

Prenatal diagnosis of congenital heart disease (CHD), the most common fetal abnormality, can greatly improve neonatal outcomes [1]. However, conducting and interpreting fetal cardiac ultrasound requires a high level of technical skill, and diagnostic accuracy commonly depends on the examiner’s experience, resulting in a relatively low prenatal diagnostic rate of only 40–50% [2,3]. One study performing quality assessment of cardiac ultrasound images obtained during the second-trimester standard anomaly scan of 92 fetuses with severe CHD found that the main reasons why CHDs were missed during prenatal ultrasound screening were inadequate magnification, poor image quality, and incomplete examination [4].

Recently, groundbreaking advances in the field of artificial intelligence (AI) have resulted in its introduction in many medical research and clinical settings [5,6,7,8,9]. When the amount of medical image data available is limited, zero-shot and few-shot learning with foundation models are options for building models [10,11]. AI technologies have been recognized as valuable approaches for standardizing examinations and improving both the efficiency and accuracy of ultrasound diagnosis [12,13]. AI features can handle several important tasks, including image acquisition, image optimization, quality control, automated measurement of biometric parameters, and anomaly detection [14,15,16,17,18]. These applications can thus reduce inter- and intra-examiner variability, save time, and improve the accuracy of ultrasound examinations [19,20]. Some vendors have therefore incorporated AI-based automated image acquisition into ultrasound machines, with these machines now able to automatically detect and classify standard cardiac views from fetal heart videos and automatically highlight them on a screen such that they can be easily recognized by the examiner. This AI feature can help to check the image quality and ensure that all key cardiac views are captured. Shadows are among the characteristic issues of ultrasound examinations, which must be considered for image optimization and quality control [21]. To overcome this issue, we propose an autoencoding structure that estimates the shadowed area and its intensities [22]. The model splits an input image into shadow and content images using its encoder and decoder, respectively. These were then combined to reconstruct the input. By generating synthetic shadows based on relatively domain-specific knowledge of ultrasound images, the model can be trained using unlabeled data. As a candidate for clinical applications, examiners can evaluate whether the currently acquired ultrasound imaging is suitable for real-time diagnosis. In the case of low image quality, rescanning can be performed in the same examination time. Some vendors have also incorporated functions that automatically measure fetal CTAR and the cardiac axis into ultrasound machines.

Arnaout et al. built an AI model for CHD detection using 1326 fetal cardiac screening videos, including 400 cases of major CHD [8]. The AI was trained to automatically recognize five standard cardiac views and determine whether each view was normal or abnormal. The AI model could detect major CHDs with expert-level accuracy (AUC 0.99, sensitivity 95%, specificity 96%) and combine those results to make a final judgment as to whether the heart is normal or has a CHD. We have also previously discussed the AI-based automated detection of 18 cardiac substructures following a sweep scan of an ultrasound probe from the abdomen to the great vessels [23]. The novelty of this system lies in the display of time-series information using barcodes and detection rate graphs. Any deviation from the information displayed for normal fetuses was flagged as a suspicious abnormality and brought to the examiner’s attention. In July 2024, our AI system that supports fetal cardiac ultrasound screening received regulatory approval from the Pharmaceuticals and Medical Devices Agency in Japan (approval number: 30600BZX00155000).

Despite the widespread use of AI technologies to improve the prenatal detection rate of CHDs, further improvements and innovations are required to improve prenatal ultrasound diagnosis of severe CHD, which is often difficult to detect. Total anomalous pulmonary venous connection (TAPVC) is a cardiac anomaly in which all pulmonary veins drain directly into the right atrium or systemic venous system. TAPVC accounts for 2% of live births from CHD [24]. Although TAPVC is a severe CHD requiring early postnatal treatment, the prenatal diagnosis rate remains relatively low [25]. To improve the accuracy of the prenatal diagnosis of TAPVC, it is essential to detect the connection between the pulmonary venous (PV) and the left atrium (LA). Conventional two-dimensional (2D) ultrasound image processing using sweep scanning makes it difficult to integrate information between frames. Although three-dimensional (3D) and four-dimensional (4D) ultrasound examinations using commercially available ultrasound equipment may improve the prenatal diagnosis of TAPVC, there is a high technical barrier for unskilled examiners, with expert experience generally required [26]. To address these issues, we formulated the following research questions (RQs):

- RQ1: Can PV-LA connections be better visualized using conventional 2D ultrasound videos?
- RQ2: How can PV-LA connections be quantitatively evaluated?
- RQ3: Does our method contribute to improving TAPVC screening performance?

In this study, we attempt to automatically visualize and detect PV-LA connections using 3D segmentation and reconstruction from 2D sweep-scanning ultrasound videos. Furthermore, we propose novel quantitative evaluation methods for PV-LA connections to classify normal connections and TAPVC. Our method should make it easier for unskilled examiners to capture the PV-LA connection and contribute to improving the accuracy of TAPVC screening.

## 2. Materials and Methods

### 2.1. Data Preparation

A total of 106 women with singleton pregnancies were enrolled and underwent fetal cardiac ultrasound screening at Showa Medical University Hospital (Tokyo, Japan). The dataset included 100 normal fetuses and six fetuses with total anomalous pulmonary venous connection (TAPVC). The mean (range) gestational age at examination was 20 (19–30) weeks for normal cases and 26 (20–33) weeks for TAPVC cases. Ultrasound videos were obtained by expert sonographers and obstetricians under direct supervision of experts using commercially available ultrasound platforms (Voluson^®^ E8 or E10; GE Healthcare, Chicago, IL, USA) in accordance with established guidelines [27]. Each recording comprised sequential cross-sectional cine images from the stomach level through the heart to the vascular arches, predominantly in the apical view (Supplementary Video S1).

### 2.2. Data Preprocessing and Augmentation

**Figure 1:**
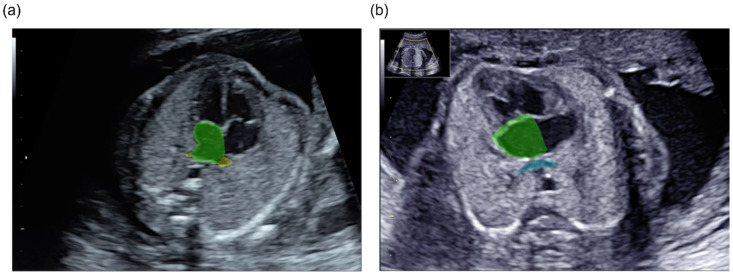
The annotated labels of the ground truth of the PV and LA in the four-chamber view of a normal case (**a**) and a TAPVC case (**b**). The LA is presented in green, the normal PV in yellow (**a**), and a confluent vein behind the LA in light blue (**b**).

The expert annotated the PV and LA pixel-by-pixel in each video, including all frames of the sequential cross-sections from the level of the four-chamber views (4CVs) to the left ventricular outflow tract (Figure 1). The expert was a clinician with board certification as a fetal cardiac ultrasound specialist. Training was conducted using 7079 randomly assigned images from 100 videos of 94 normal cases. The data were assigned for training and validation in a 4:1 ratio, with no overlap across the training, validation, or test datasets. Cross-validation was used to select the best model for each 3D segmentation model. As an independent test dataset, 1033 images from 12 videos of six normal cases and 949 images from 12 videos of six TAPVC cases were used.

Data preprocessing was applied using an open-source MONAI library as follows. First, all images were resized to the spatial size (128 × 128 × 160 pixels). Then, we performed intensity normalization using ScaleIntensityRanged to linearly transform the input intensities to the range of [0, 255] to [0, 1], and clipped the values. To extract the training patches, we used RandCropByPosNegLabeld, setting it to include an equal number of positive and negative examples (pos = 1, neg = 1), and generated two patches per sample. Owing to the limited amount of available data, we conducted data augmentation using the MONAI library. The augmentation pipeline comprises random cropping guided by positive and negative label sampling, spatial transformations (random flipping, zooming, and elastic deformation), and intensity-based transformations (Gaussian noise, bias-field augmentation, and random intensity scaling and shifting) (Supplementary Figure S1). For spatial transformations, we used RandFlipd, applying random flips along spatial axes 1 and 2 with a probability of 0.2, respectively. The zoom factor was randomly varied in the range of [0.9, 1.1] by RandZoomd, with a probability of application of 0.5. Rand3DElasticd was used for elastic deformation, with the standard deviation (sigma) of Gaussian smoothing set to a random range of [4.0, 6.0] and the magnitude of displacement set to a random range of [20.0, 80.0]. For intensity-based transformations, RandScaleIntensityd was applied with a probability of 0.1 and the scaling factor was set to 0.1. The intensity offset was randomly varied in the range of 0.1 using RandShiftIntensityd, with an application probability of 0.1. RandGaussianNoised was applied with a probability of 0.5 to add noise. Additionally, RandBiasField was used with a probability of 0.2 to simulate brightness non-uniformity. Data augmentation was applied only to the training data, not to the validation or test data. In addition, all evaluations of the test data were performed using the original data, and caution was exercised to avoid any bias in the evaluation due to data augmentation.

### 2.3. Three-Dimensional Segmentation

#### 2.3.1. Three-Dimensional Segmentation Models

The following state-of-the-art 3D medical segmentation models were applied to segment the PV-LA aggregate area: SegResNet, Swin UNETR, MedNeXt, and SegFormer3D. SegResNet is an encoder–decoder-based model, which uses repeated ResNet blocks with instance normalization, and deep supervision in the decoder branch [28]. Swin UNETR consists of a hierarchical Swin transformer-based encoder and fully convolutional neural network-based decoder, which realizes a highly efficient network structure with linear computational complexity relative to the image size by limiting the range of self-attention to a fixed-size window [29]. MedNeXt is a Transformer-inspired large kernel model that iteratively increases kernel sizes by upsampling small kernel networks to prevent performance saturation in limited medical data [30]. SegFormer3D is a hierarchical Transformer that calculates attention across multiscale volumetric features [31].

We used the default network architecture and hyperparameter settings of each model provided in the MONAI framework for SegResNet, Swin UNETR, and MedNeXt. For SegFormer 3D, we acquired the implementation from a public GitHub repository and trained it using the default hyperparameters defined in the repository. No special hyperparameter tuning was performed in any of the models. The rationale for adopting these settings is to ensure fairness and reproducibility in comparisons across the models by using standard architecture based on public implementations. All models were laid out to accept grayscale 3D volume data as input and process 4D data consisting of depth (time direction), height, and width. The number of trainable parameters for each model was approximately 1.18 million for SegResNet, 15.7 million for Swin UNETR, 6.59 million for MedNeXt, and 4.49 million for SegFormer3D (Supplementary Table S1).

#### 2.3.2. Training Environment and Implementation Details

All models in this study were trained based on the following computing environment and settings. The batch size during training and validation was 1 and 2, respectively, and the number of training epochs was 300. The initial learning rate was set to 0.001, the minimum learning rate to 1 × 10^−6^, and the weight decay was set to 0.001. AdamW was used as the optimization method. The learning rate scheduler combined warm-up and cosine annealing, linearly increasing the learning rate with LinearLR for the first 10 epochs, followed by reduction to the minimum learning rate with CosineAnnealingLR for the remaining epochs. Implementation was performed using Python 3.13.5, with the following main libraries: PyTorch (version 2.9.1+cu128), MONAI (version 1.5.1), NumPy (version 2.2.6), and SimpleITK (version 2.5.2). All experiments were performed on a GPU environment equipped with one NVIDIA Tesla V100 SXM3 graphics card (32 GB memory) (NVIDIA, Santa Clara, CA, USA).

#### 2.3.3. Segmentation Performance Evaluation

The Dice coefficient was applied to measure the accuracy of the segmentation model based on true positive (TP), false positive (FP), and false negative (FN) values, and was calculated as (1):

(1) Dice = 2TP/(2TP + FP + FN).

Values of the Dice coefficients range from 0 to 1, where values closer to 1 indicate better segmentation performance. To evaluate the accuracy of the four segmentation models, the mean dice values (mDice) were calculated for each ultrasound video using the ground-truth labels and inference results.

The 95th percentile of the Hausdorff distance (HD95) was also calculated. The Hausdorff distance was defined as (2):

(2) $$HD\left(P,G\right)=\max\left\{\underset{p\in P}{\max}\;\underset{g\in G}{\min}d(p,g),\underset{g\in G}{\max}\;\underset{p\in P}{\min}d(g,p)\right\}$$

where *P* and *G* denote the sets of surface points of the predicted segmentation and the ground-truth, respectively, and d(⋅,⋅) denotes the Euclidean distance between two surface points. HD95 was computed by taking the 95th percentile of the surface-to-surface distances, thereby reducing the sensitivity to outliers. Values of HD95 are 0 or above, where values approximating 0 indicate better segmentation performance.

(3) $$HD_{95}\left(P,G\right)=percentile_{95}\left(D\right).$$

where

$$D=D_{P\to G}\cup D_{G\to P}\;D_{P\to G}=\{\underset{g\in G}{\min}d\left(p,g\right)|p\in P\}\;D_{G\to P}=\{\underset{p\in P}{\min}d\left(g,p\right)|g\in G\}$$

### 2.4. Quantitative Evaluation of the PV-LA Connection

#### 2.4.1. PV-LA Distance (PLD)

The PV and LA are 3D binary masks of size H × W × D with a range of {0, 1}; *K* is a set of slices in which there is at least one pixel with PV=1 and one pixel with LA=1 in each slice *k* among the depth-direction index k∈{1,…,D} . For each k∈K , it is defined as the minimum Euclidean distance between the pixel with PV=1 and the pixel with LA=1 in the same slice (Supplementary Figure S2). This distance set *D* is defined as $\left\{d_{k}||k\in K\right\}$ . Furthermore, $t_{q}$ is the quantile corresponding to the bottom q% in the distance set *D*. $D_{q}$ , which represents the subset of the distances less than $t_{q}$ , was calculated as (4):

(4) $$D_{q}=\left\{\;d\;\right|\;d\in D,\;d\leq t_{q}\}.$$

Subsequently, the mean values of $D_{q}$ were calculated as the PLD:

(5) $$PLD=\frac{1}{\left|D_{q}\right|}\sum_{d\in D_{q}}d.$$

**Figure 2:**
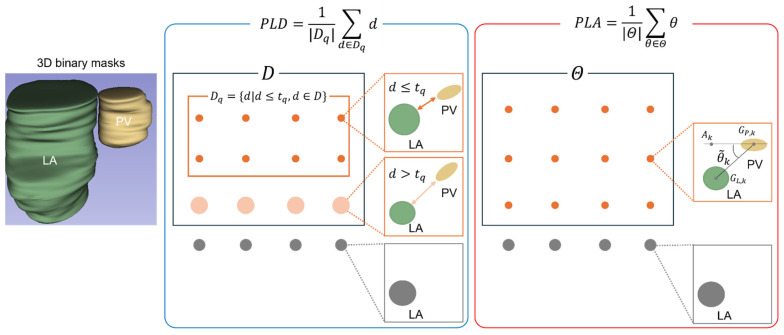
Schema showing processing of the subset for the PLD and PLA calculation based on 3D binary masks. For the quantitative evaluation of the PV-LA connection, the mean values of the shortest PV-LA distance (PLD) and the major axis angle (PLA) in each video were calculated.

The units of PLD are pixels. Because there is no situation in which the PV and LA are simultaneously zero, the minimum PLD is 1.00, indicating a PV-LA connection. The larger the PLD, the greater the discrepancy between the PV and LA. A larger PLD indicated that the PV and LA were more divergent (Figure 2).

#### 2.4.2. PV-LA Angle (PLA)

Similarly to the PLD, a set *K* is used. $G_{P,k},G_{L,k}$ are the centers of gravity of the PV and LA, respectively, in each slice *k*; $A_{k}$ is any point on the major axis of the ellipse approximating the PV that is different from $G_{P,k}$ ; and the angle between vectors $G_{P,k}G_{L,k}$ and $G_{P,k}A_{k}$ was defined as $\theta_{k}\in [0,180{}^{\circ}]$ and ${\tilde{\theta }}_{k}=\min\left(\theta_{k},180{}^{\circ}-\theta_{k}\right)$ (Supplementary Figure S3). Θ represents the subset of the angles obtained from all slices in which there are both the PV and LA. The PLA, defined as the mean values of Θ , was calculated as (6):

(6) $$PLA=\frac{1}{\left|\Theta \right|}\sum_{\theta \in \Theta }\theta .$$

The unit of PLA is degrees. The PLA ranged from 0 to 90.0. The larger the PLA, the greater the deviation between the PV and LA, indicating that the PV approaches horizontality relative to the LA (Figure 2).

### 2.5. Screening Performance Comparison

To assess the screening performance for TAPVC using PLD and PLA, receiver operating characteristic (ROC) curve analysis was performed based on the mean PLD and PLA values. The areas under the ROC curves (AUCs) were compared across the four segmentation models using their arithmetic means. Higher AUC values indicate better discrimination between normal and TAPVC cases.

## 3. Results

### 3.1. Three-Dimensional Visualization and Detection of the PV-LA Connection

**Figure 3:**
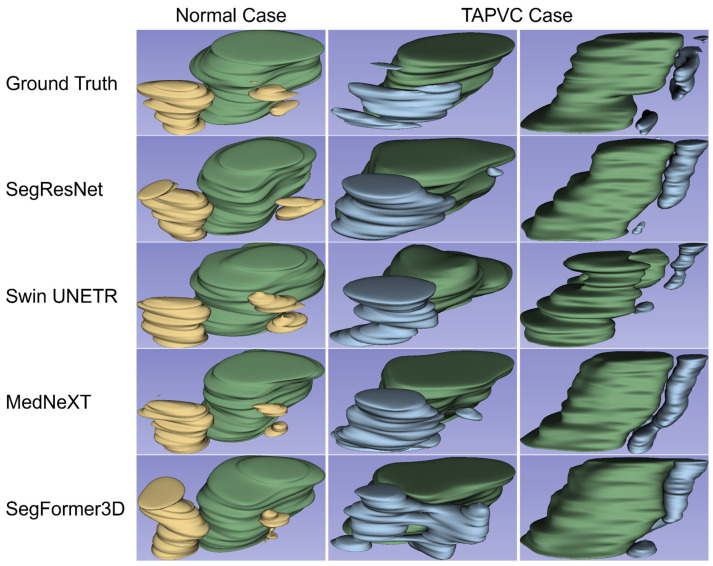
Representative 3D segmentation and reconstruction images in the PV-LA connection for the four models. The images on the top row contain annotated labels of the ground truth. One horizontal row presents the segmentation results for each model. The images in the left column show a normal case, the middle column shows a TAPVC case viewed from the same angle as the normal case, and the right column shows the same TAPVC case viewed from the side. The LA is presented in green, while the PVs are shown in yellow and light blue in the normal and TAPVC case, respectively.

Figure 3 presents the 3D segmentation and reconstruction images of the PV-LA connection using ground-truth labels and the four models. Overall, these models demonstrated sufficient performance in visualizing and detecting PV-LA connections in normal cases. In contrast, the PV and LA diverged, and a confluent vein was present behind the LA in TAPVC cases.

Table 1 presents a comparison of the segmentation results of the four models based on mDice and HD95 using the independent test data of normal and TAPVC cases. The PV-LA connections of the normal cases were segmented with comparable quality for each model. The segmentation performance of the TAPVC cases was inferior to that of the normal cases. SegFormer3D showed the best performance in TAPVC cases.

### 3.2. Calculation and Evaluation of the PLD and PLA

**Figure 4:**
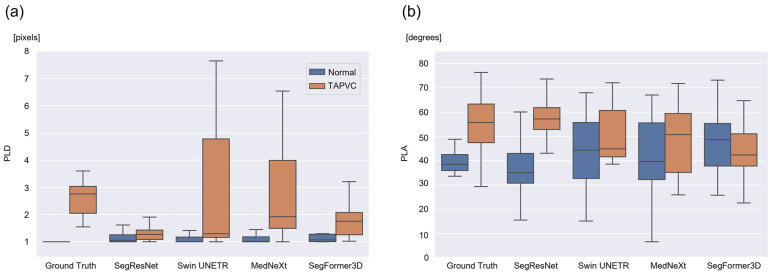
Distribution of the PLD (**a**) and PLA (**b**) for each model. According to the ground truth, both the PLD and PLA tended to be higher in TAPVC cases than those in normal cases. Each model was evaluated using suitable values of the PLD and PLA, and the larger discrepancy in these indices between the normal and TAPVC cases. PLD, PV-LA distance; PLA, PV-LA angle.

Using the ground-truth labels of normal cases, the mean ± SD range for the PLD and PLA was defined as standard values of 1.00 ± 0.011 and 37.8 ± 7.72, respectively. In TAPVC cases, the mean ± SD range for the PLD and PLA was defined as standard values of 2.64 ± 0.667 and 55.1 ± 14.5, respectively. Both PLD and PLA tended to be higher in TAPVC cases than in normal cases, consistent with the clinical findings. Table 2 presents the calculated PLD and PLA values for each model. First, the PLD of normal cases should approximate a minimum of 1.00, indicating a PV-LA connection. Based on the above-mentioned standard values and MAE for the PLD, SegResNet and MedNeXt were considered suitable for normal cases. Next, the greater the difference in PLD between normal and TAPVC cases, the better the classification performance. Considering this point, MedNeXt yielded the most acceptable results (Figure 4a). Regarding PLA, SegResNet, Swin UNETR, and MedNeXt showed similar trends as the standard values of the normal/TAPVC cases. In contrast, SegFormer3D showed the opposite trend. Considering the larger discrepancy in PLA between normal and TAPVC cases, SegResNet yielded the best results (Figure 4b).

### 3.3. TAPVC Screening Performance Using the PLD and PLA

**Figure 5:**
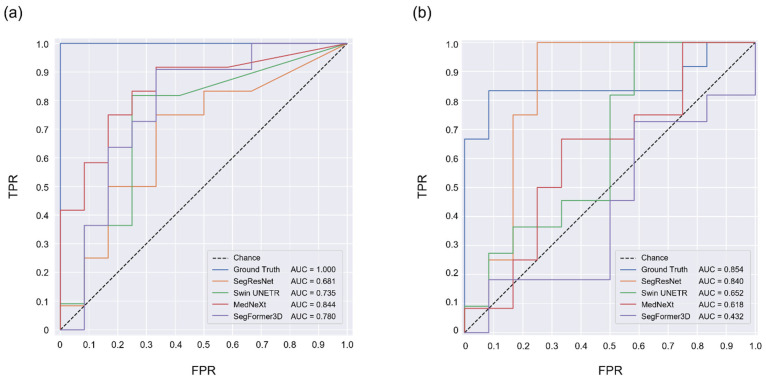
Performance of TAPVC screening based on the PLD (**a**) and PLA (**b**). The ROC curves present the screening performances of the ground-truth labels and individual models. The mean values of the AUCs are reported in the legends. ROC, receiver operating characteristic; AUC, area under the receiver operating characteristic curve; TPR, true-positive rate; FPR, false-positive rate.

The ground-truth labels showed the highest performance in classifying the normal and TAPVC cases using PLD and PLA, a mean AUC of 1.000 and 0.854, respectively. In the screening performance comparison of the 3D segmentation models using PLD, the mean AUCs of SegResNet/Swin UNETR/MedNeXt/SegFormer3D were 0.681/0.735/0.844/0.780, respectively. MedNeXt-PLD achieved the best performance for TAPVC screening based on the PLD (Figure 5a). Regarding the screening performance comparison of these models based on the PLA, the mean AUCs of SegResNet/Swin UNETR/MedNeXt/SegFormer3D were 0.840/0.652/0.618/0.432, respectively. Therefore, SegResNet-PLA achieved the best performance for TAPVC screening based on the PLA, which was comparable to that of the ground-truth labels (Figure 5b).

## 4. Discussion

TAPVC is a potentially lethal CHD that occurs when the PV does not properly connect to the LA, resulting in mixing of pulmonary and systemic blood [24]. Little is known about the mechanisms underlying venous patterning and development of TAPVC. During normal embryonic development, the primordial PV opens into the primitive LA at approximately the fifth week of gestation and is subsequently absorbed into the LA by the sixth week, resulting in the formation of the PV orifices within the LA. In contrast, in TAPVC, the PVs fail to undergo normal absorption into the LA and instead establish anomalous connections with the systemic venous circulation [32]. The secreted guidance molecule, semaphorin 3d, was reported to be a key cue for PV patterning and in vivo experimental evidence for an alternative developmental model to explain abnormal PV connections has previously been provided [33].

Using conventional 2D ultrasound video, the small size and short imaging time of the PV make ultrasound screening of TAPVC difficult (Supplementary Video S1). Our previous study demonstrated a relatively low mean accuracy for PV detection in 18 cardiac substructures on fetal cardiac 2D ultrasound video, even with AI. Moreover, although the PV and LA could be detected individually, the PV-LA connection could not be detected [23]. Indeed, it is nearly impossible for unskilled examiners to capture PV–LA connections, with this process requiring significant expertise. From a clinical imaging perspective, prenatal detection of TAPVC using fetal cardiac ultrasound relies primarily on the direct confirmation of normal PV connections to the LA on the 4CV. When direct visualization is inconclusive, several indirect ultrasound findings may raise suspicion of TAPVC, including the presence of a vascular confluence posterior to the LA, enlargement of the retroatrial space, a smooth posterior left atrial wall, dilation of the superior vena cava or coronary sinus, and visualization of abnormal vessels such as a vertical vein, which are not observed normally [34,35]. Based on these indirect ultrasound findings, the post-left atrium space (PLAS) index and left-atrial posterior-space-to-diagonal (LAPSD) ratio have been proposed as imaging markers [36,37,38]. However, despite advances in ultrasound machines and molecular biology analyses, the prenatal diagnosis rate of TAPVC remains suboptimal [25].

In the present study, first, the 3D segmentation models demonstrated sufficient performance in visualizing and detecting PV-LA connections in normal cases. The PVs can be distinguished and recognized as draining into the LA. Reconstructing 2D information from ultrasound videos enabled accurate 3D segmentation of PV-LA connections. Three-dimensional segmentation was superior to 2D segmentation in visualizing the relationship between the small PVs and LA. These findings answer RQ1. No significant differences were observed in the mDice and HD95 values among the four models. These indicators may plateau due to several factors, including the fetal heartbeat. In contrast, in TAPVC cases, the PV and LA diverged, and a confluent vein was present behind the LA. These visual results reflect the clinical condition of patients with TAPVC. The values of mDice and HD95 in the TAPVC cases were lower than those in the normal cases. Although SegFormer3D showed the best performance in TAPVC cases, it was insufficient. These models were trained using only a dataset of normal cases, which may result in poor segmentation performance when dealing with abnormal variations in TAPVC.

Next, we propose two novel indices, PLD and PLA, for the quantitative evaluation of the PV-LA connection, which require the calculation of the mean shortest PV-LA distance (PLD) and major axis angle (PLA). The standard values of these indices were confirmed using the ground-truth labels of the normal and TAPVC cases. The PLD of normal cases was close to a minimum of 1.00, indicating a PV-LA connection. Both the PLD and PLA tended to be higher in TAPVC cases than in normal cases. These results reflect the clinical status and mechanism of TAPVC in the PV-LA connection. Subsequently, we assessed the distribution of the mean ± SD and MAE values of the PLD and PLA in normal and TAPVC cases. Considering the suitable values of the PLD, and the larger discrepancy in the PLD between normal and TAPVC cases, MedNeXt was the most acceptable among the four models. Regarding PLA, SegResNet, Swin UNETR, and MedNeXt were all found to be comparable, while SegFormer3D showed the opposite trend. Among the three models, SegResNet yielded the largest discrepancy in the PLA between normal and TAPVC cases. These results address RQ2.

Finally, the TAPVC screening performances of the four models were compared for classifying normal and TAPVC cases using the PLD and PLA. The ground-truth labels performed the best for both PLD and PLA, achieving a mean AUC of 1.000, especially for the PLD. From the results of the mean AUCs, MedNeXt-PLD and SegResNet-PLA achieved the best performance in detecting TAPVC. SegResNet-PLA showed a mean AUC of 0.840, comparable to 0.854 for the ground-truth labels. These findings indicate that MedNeXt-PLD might require further improvement of 3D segmentation, while SegResNet-PLA was able to capture the actual PV-LA connections well. In this context, we can answer RQ3, i.e., combining direct confirmation of PV-LA connections with quantitative evaluation of PV-LA spatial indices, including distance and angular orientation, enhances the prenatal detection of TAPVC.

To the best of our knowledge, no related study has been reported to date on 3D segmentation and reconstruction of the PV-LA connection using AI in fetal cardiac ultrasound videos. Although the target patient age and the medical imaging domain were different, Li et al. proposed an automatic segmentation method for the PV and LA in low-dose CT images for preoperative evaluation and planning of postnatal TAPVC [39]. Wang et al. reported that several 2D segmentation models achieved good diagnostic accuracy for TAPVC based on the PLAS index using still ultrasound images in the 4CVs [40]. However, the PLAS index was calculated from the segmentation of the epicardium and descending aorta, not by detecting PV-LA connections directly. In addition, the 4CV frames were manually selected from the videos in their study. In clinical practice, the best frame selection also depends on the skill levels of the examiner.

Our method integrates information between the frames of 2D sweep-scan ultrasound videos to provide 3D visualization, allowing unskilled examiners to capture the PV-LA connection. Furthermore, this approach expands on previously reported qualitative sonographic criteria by incorporating objective spatial indices, aligns closely with the embryological mechanisms underlying TAPVC, and provides a rational framework for interpreting its characteristic prenatal ultrasound findings.

### Limitations

This study has several limitations that should be considered. Firstly, the training data for our models were limited, as only a few fetal cardiac ultrasound videos of TAPVC are available. Further, our model was trained using a dataset of normal cases only; as such, they must be further tested to better detect abnormal variations in TAPVC. Considering the low incidence of TAPVC, a multicenter study should be conducted to collect sufficient data. Second, the segmentation of PV-LA connections may stagnate owing to several factors, such as the performance limitations of the current model and fetal heartbeat. To increase the reliability of our method in clinical practice, a prospective multicenter study focusing on the performance of TAPVC screening should be conducted. Third, the fetal cardiac ultrasound videos analyzed in this study were predominantly acquired by experts. This is important as video quality varies depending on the skill level of the examiners. To support unskilled examiners, the quality control of the input videos must be addressed using other techniques. Finally, we only used videos of the ultrasound machines from one vendor. To better facilitate the clinical application of our method, its compatibility with other existing ultrasound equipment and domain shifts should be considered.

## 5. Conclusions

Overall, in the present study, we developed a new method to allow the 3D visualization and automatic detection of the PV and LA in fetal cardiac ultrasound videos. We also proposed two novel indices, the PLD and PLA, for the quantitative evaluation of the relationship between the PV and LA. Our findings demonstrate that these models provide reliable visualization of PV-LA connections and offer promising screening performance for TAPVC, with MedNeXt-PLD and SegResNet-PLA achieving mean AUC values of 0.844 and 0.840, respectively. This method will make it easier for unskilled examiners to understand the PV-LA connection and is expected to improve the accuracy of TAPVC screening. In the near future, AI-assisted fetal cardiac screening should extend its clinical use and contribute to improving the prenatal ultrasound diagnostic rate of CHDs.

## Figures and Tables

**Table 1:** Segmentation performance of the PV-LA connection.

Model	Normal Cases		TAPVC Cases	
	mDice	HD95	mDice	HD95
SegResNet	0.784	5.12	0.443	17.7
Swin UNETR	0.738	9.72	0.323	28.8
MedNeXt	0.765	6.36	0.448	23.3
SegFormer3D	0.713	8.11	0.563	8.49

mDice, means value of the Dice coefficient; HD95, 95% Hausdorff distance.

**Table 2:** The PLD and PLA values calculated for each model.

Model	Normal Cases				TAPVC Cases			
	PLD		PLA		PLD		PLA	
	Mean ± SD	MAE	Mean ± SD	MAE	Mean ± SD	MAE	Mean ± SD	MAE
SegResNet	1.19 ± 0.281	0.185	38.6 ± 15.8	8.24	1.31 ± 0.289	1.33	57.4 ± 9.26	14.4
Swin UNETR	1.78 ± 2.43	0.779	43.2 ± 16.0	9.71	4.22 ± 6.10	3.39	51.1 ± 12.0	15.9
MedNeXt	1.23 ± 0.420	0.223	40.6 ± 18.8	9.10	5.15 ± 9.35	3.81	48.2 ± 15.7	13.4
SegFormer3D	1.94 ± 2.75	0.941	47.3 ± 13.0	10.7	2.07 ± 1.37	1.35	43.4 ± 13.2	14.8

PLD, PV-LA distance; PLA, PV-LA angle; SD, standard deviation; MAE, mean absolute error.

## Data Availability

All data supporting this study are contained within the article and Supplementary Materials.

## Supplementary Materials

The following supporting information can be downloaded from: https://www.mdpi.com/article/10.3390/bioengineering13010100/s1, Figure S1: Representative augmented images after data augmentation; Figure S2: Schema of PV-LA connections for the PLD calculation of a normal case and a TAPVC case; Figure S3: Schema of PV-LA connections for the PLA calculation of a normal case and a TAPVC case; Table S1: Default hyperparameters for each model; Video S1: Fetal cardiac ultrasound screening.

## References

[1] Qiu X., Weng Z., Liu M., Chen X., Wu Q., Ling W., Ma H., Huang H., Lin Y. (2020). Prenatal diagnosis and pregnancy outcomes of 1492 fetuses with congenital heart disease: Role of multidisciplinary-joint consultation in prenatal diagnosis. Sci. Rep..

[2] Donofrio M.T., Moon-Grady A.J., Hornberger L.K., Copel J.A., Sklansky M.S., Abuhamad A., Cuneo B.F., Huhta J.C., Jonas R.A., Krishnan A. (2014). Diagnosis and treatment of fetal cardiac disease: A scientific statement from the American Heart Association. Circulation.

[3] Matsui H., Hirata Y., Inuzuka R., Hayashi T., Nagamine H., Ueda T., Nakayama T. (2021). Initial national investigation of the prenatal diagnosis of congenital heart malformations in Japan-Regional Detection Rate and Emergency Transfer from 2013 to 2017. J Cardiol.

[4] van Nisselrooij A.E.L., Teunissen A.K.K., Clur S.A., Rozendaal L., Pajkrt E., Linskens I.H., Rammeloo L., van Lith J.M.M., Blom N.A., Haak M.C. (2020). Why are congenital heart defects being missed?. Ultrasound Obstet. Gynecol..

[5] Yamada Y., Kobayashi M., Shinkawa K., Bilal E., Liao J., Nemoto M., Ota M., Nemoto K., Arai T. (2025). Utility of synthetic musculoskeletal gaits for generalizable healthcare applications. Nat. Commun..

[6] Campanella G., Hanna M.G., Geneslaw L., Miraflor A., Werneck Krauss Silva V., Busam K.J., Brogi E., Reuter V.E., Klimstra D.S., Fuchs T.J. (2019). Clinical-grade computational pathology using weakly supervised deep learning on whole slide images. Nat. Med..

[7] Yamada M., Saito Y., Imaoka H., Saiko M., Yamada S., Kondo H., Takamaru H., Sakamoto T., Sese J., Kuchiba A. (2019). Development of a real-time endoscopic image diagnosis support system using deep learning technology in colonoscopy. Sci. Rep..

[8] Arnaout R., Curran L., Zhao Y., Levine J.C., Chinn E., Moon-Grady A.J. (2021). An ensemble of neural networks provides expert-level prenatal detection of complex congenital heart disease. Nat. Med..

[9] Rao V.M., Hla M., Moor M., Adithan S., Kwak S., Topol E.J., Rajpurkar P. (2025). Multimodal generative AI for medical image interpretation. Nature.

[10] Wang Z., Yang Y., Chen Y., Yuan T., Sermesant M., Delingette H., Wu O. (2024). Mutual Information Guided Diffusion for Zero-Shot Cross-Modality Medical Image Translation. IEEE Trans. Med. Imaging.

[11] Wang W., Li Y., Lu K., Zhang J., Chen P., Yan K., Wang B. (2024). Medical Tumor Image Classification Based on Few-Shot Learning. IEEE/ACM Trans. Comput. Biol. Bioinform..

[12] Fiorentino M.C., Villani F.P., Di Cosmo M., Frontoni E., Moccia S. (2023). A review on deep-learning algorithms for fetal ultrasound-image analysis. Med. Image Anal..

[13] Liu B., Chang H., Yang D., Yang F., Wang Q., Deng Y., Li L., Lv W., Zhang B., Yu L. (2023). A deep learning framework assisted echocardiography with diagnosis, lesion localization, phenogrouping heterogeneous disease, and anomaly detection. Sci. Rep..

[14] Baumgartner C.F., Kamnitsas K., Matthew J., Fletcher T.P., Smith S., Koch L.M., Kainz B., Rueckert D. (2017). SonoNet: Real-Time Detection and Localisation of Fetal Standard Scan Planes in Freehand Ultrasound. IEEE Trans. Med. Imaging.

[15] Abdi A.H., Luong C., Tsang T., Allan G., Nouranian S., Jue J., Hawley D., Fleming S., Gin K., Swift J. (2017). Automatic Quality Assessment of Echocardiograms Using Convolutional Neural Networks: Feasibility on the Apical Four-Chamber View. IEEE Trans. Med. Imaging.

[16] Slimani S., Hounka S., Mahmoudi A., Rehah T., Laoudiyi D., Saadi H., Bouziyane A., Lamrissi A., Jalal M., Bouhya S. (2023). Fetal biometry and amniotic fluid volume assessment end-to-end automation using Deep Learning. Nat. Commun..

[17] Aoyama R., Komatsu M., Harada N., Komatsu R., Sakai A., Takeda K., Teraya N., Asada K., Kaneko S., Iwamoto K. (2024). Automated assessment of the pulmonary artery-to-ascending aorta ratio in fetal cardiac ultrasound screening using artificial intelligence. Bioengineering.

[18] Yeo L., Luewan S., Romero R. (2018). Fetal Intelligent Navigation Echocardiography (FINE) Detects 98% of Congenital Heart Disease. J. Ultrasound Med..

[19] Bridge C.P., Ioannou C., Noble J.A. (2017). Automated annotation and quantitative description of ultrasound videos of the fetal heart. Med. Image Anal..

[20] Lam-Rachlin J., Punn R., Behera S.K., Geiger M., Lachaud M., David N., Garmel S., Fox N.S., Rebarber A., DeVore G.R. (2026). Use of Artificial Intelligence-Based Software to Aid in the Identification of Ultrasound Findings Associated With Fetal Congenital Heart Defects. Obstet. Gynecol..

[21] Meng Q., Sinclair M., Zimmer V., Hou B., Rajchl M., Toussaint N., Oktay O., Schlemper J., Gomez A., Housden J. (2019). Weakly Supervised Estimation of Shadow Confidence Maps in Fetal Ultrasound Imaging. IEEE Trans. Med. Imaging.

[22] Yasutomi S., Arakaki T., Matsuoka R., Sakai A., Komatsu R., Shozu K., Dozen A., Machino H., Asada K., Kaneko S. (2021). Shadow Estimation for Ultrasound Images Using Auto-Encoding Structures and Synthetic Shadows. Appl. Sci..

[23] Komatsu M., Sakai A., Komatsu R., Matsuoka R., Yasutomi S., Shozu K., Dozen A., Machino H., Hidaka H., Arakaki T. (2021). Detection of cardiac structural abnormalities in fetal ultrasound videos using deep learning. Appl. Sci..

[24] Abuhamad A.Z., Chaoui R. (2022). A Practical Guide to Fetal Echocardiography: Normal and Abnormal Hearts.

[25] Paladini D., Pistorio A., Wu L.H., Meccariello G., Lei T., Tuo G., Donarini G., Marasini M., Xie H.N. (2018). Prenatal diagnosis of total and partial anomalous pulmonary venous connection: Multicenter cohort study and meta-analysis. Ultrasound Obstet. Gynecol..

[26] Bravo-Valenzuela N.J.M., Peixoto A.B., Araujo Júnior E. (2021). Prenatal diagnosis of total anomalous pulmonary venous connection: 2D and 3D echocardiographic findings. J. Clin. Ultrasound.

[27] Carvalho J.S., Axt-Fliedner R., Chaoui R., Copel J.A., Cuneo B.F., Goff D., Gordin Kopylov L., Hecher K., Lee W., Moon-Grady A.J. (2023). ISUOG practice guidelines (updated): Fetal cardiac screening. Ultrasound Obstet. Gynecol..

[28] Myronenko A. 3D MRI brain tumor segmentation using autoencoder regularization. Proceedings of the International MICCAI Brainlesion Workshop.

[29] Hatamizadeh A., Nath V., Tang Y., Yang D., Roth H.R., Xu D. Swin unetr: Swin transformers for semantic segmentation of brain tumors in mri images. Proceedings of the International MICCAI Brainlesion Workshop.

[30] Roy S., Koehler G., Ulrich C., Baumgartner M., Petersen J., Isensee F., Jäger P.F., Maier-Hein K.H. MedNeXt: Transformer-Driven Scaling of ConvNets for Medical Image Segmentation. Proceedings of the Medical Image Computing and Computer Assisted Intervention—MICCAI 2023.

[31] Perera S., Navard P., Yilmaz A. Segformer3d: An efficient transformer for 3d medical image segmentation. Proceedings of the IEEE/CVF Conference on Computer Vision and Pattern Recognition.

[32] Kao C.C., Hsieh C.C., Cheng P.J., Chiang C.H., Huang S.Y. (2017). Total Anomalous Pulmonary Venous Connection: From Embryology to a Prenatal Ultrasound Diagnostic Update. J. Med. Ultrasound.

[33] Degenhardt K., Singh M.K., Aghajanian H., Massera D., Wang Q., Li J., Li L., Choi C., Yzaguirre A.D., Francey L.J. (2013). Semaphorin 3d signaling defects are associated with anomalous pulmonary venous connections. Nat. Med..

[34] Tongsong T., Luewan S., Jatavan P., Tongprasert F., Sukpan K. (2016). A Simple Rule for Prenatal Diagnosis of Total Anomalous Pulmonary Venous Return. J. Ultrasound Med..

[35] Chih W.L., Ko H., Chang T.Y. (2024). Prenatal Ultrasound Markers of Isolated Total Anomalous Pulmonary Venous Return and a Sequential Approach to Reach Diagnosis. J. Med. Ultrasound.

[36] Kawazu Y., Inamura N., Shiono N., Kanagawa N., Narita J., Hamamichi Y., Kayatani F. (2014). ‘Post-LA space index’ as a potential novel marker for the prenatal diagnosis of isolated total anomalous pulmonary venous connection. Ultrasound Obstet. Gynecol..

[37] Kawazu Y., Inamura N., Kayatani F., Taniguchi T. (2019). Evaluation of the post-LA space index in the normal fetus. Prenat. Diagn..

[38] Anuwutnavin S., Unalome V., Rekhawasin T., Tongprasert F., Thongkloung P. (2023). Fetal left-atrial posterior-space-to-diagonal ratio at 17-37 weeks’ gestation for prediction of total anomalous pulmonary venous connection. Ultrasound Obstet. Gynecol..

[39] Li J., Chen H., Zhu F., Wen C., Chen H., Wang L. Automatic Pulmonary Vein and Left Atrium Segmentation for TAPVC Preoperative Evaluation Using V-Net with Grouped Attention. Proceedings of the 2020 42nd Annual International Conference of the IEEE Engineering in Medicine & Biology Society (EMBC).

[40] Wang X., Yang T.Y., Zhang Y.Y., Liu X.W., Zhang Y., Sun L., Gu X.Y., Chen Z., Guo Y., Xue C. (2022). Diagnosis of fetal total anomalous pulmonary venous connection based on the post-left atrium space ratio using artificial intelligence. Prenat. Diagn..

