# Correction: Komatsu et al. Three-Dimensional Visualization and Detection of the Pulmonary Venous–Left Atrium Connection Using Artificial Intelligence in Fetal Cardiac Ultrasound Screening. *Bioengineering* 2026, *13*, 100

**DOI:** 10.3390/bioengineering13060672

**Published:** 2026-06-10

**Authors:** Reina Komatsu, Masaaki Komatsu, Katsuji Takeda, Naoaki Harada, Naoki Teraya, Shohei Wakisaka, Takashi Natsume, Tomonori Taniguchi, Rina Aoyama, Mayumi Kaneko, Kazuki Iwamoto, Ryu Matsuoka, Akihiko Sekizawa, Ryuji Hamamoto

**Affiliations:** 1 Department of Obstetrics and Gynecology, Showa Medical University School of Medicine, 1-5-8 Hatanodai, Shinagawa-ku, Tokyo 142-8666, Japanmayumikaneko@med.showa-u.ac.jp (M.K.); ryu@med.showa-u.ac.jp (R.M.); sekizawa@med.showa-u.ac.jp (A.S.); 2 AI Medical Engineering Team, RIKEN Center for Advanced Intelligence Project, 1-4-1 Nihonbashi, Chuo-ku, Tokyo 103-0027, Japan; 3 Division of Medical AI Research and Development, National Cancer Center Research Institute, 5-1-1 Tsukiji, Chuo-ku, Tokyo 104-0045, Japan; 4 Department of NCC Cancer Science, Biomedical Science and Engineering Track, Graduate School of Medical and Dental Sciences, Institute of Science Tokyo, 1-5-45 Yushima, Bunkyo-ku, Tokyo 113-8510, Japan; 5 Digital Health Platform Development Office, Healthcare Business Unit, Fujitsu Japan Ltd., 1-5 Omiya-cho, Saiwai-ku, Kawasaki 212-0014, Japan; 6 Department of Obstetrics and Gynecology, Graduate School of Medicine, The University of Tokyo, 7-3-1 Hongo, Bunkyo-ku, Tokyo 113-8655, Japan; 7 Department of Obstetrics and Gynecology, Tohoku University Graduate School of Medicine, 2-1 Seiryomachi, Aoba-ku, Sendai 980-8574, Japan

## Wrong Reference

In the original publication [1], there was a mistake in relation to the cited references:

8. Kaneko, S.; Takasawa, K.; Asada, K.; Shiraishi, K.; Ikawa, N.; Machino, H.; Shinkai, N.; Matsuda, M.; Masuda, M.; Adachi, S.; et al. Mechanism of ERBB2 gene overexpression by the formation of super-enhancer with genomic structural abnormalities in lung adenocarcinoma without clinically actionable genetic alterations. *Mol. Cancer* **2024**, *23*, 126.

should be replaced by

8. Arnaout, R.; Curran, L.; Zhao, Y.; Levine, J.C.; Chinn, E.; Moon-Grady, A.J. An ensemble of neural networks provides expert-level prenatal detection of complex congenital heart disease. *Nat. Med.* **2021**, *27*, 882–891. https://doi.org/10.1038/s41591-021-01342-5.

However, the order of references remains the same. The authors state that the scientific conclusions are unaffected. This correction was approved by the Academic Editor. The original publication has also been updated.
