# Supplementary material for: Three-Dimensional Visualization and Detection of the Pulmonary Venous–Left Atrium Connection Using Artificial Intelligence in Fetal Cardiac Ultrasound Screening
Source: Bioengineering (Basel). 2026 Jan 15;13(1):100. doi: 10.3390/bioengineering13010100 (PMC12837485; doi:10.3390/bioengineering13010100)
Supplement: Supplementary file 1 [file bioengineering-13-00100-s001.zip › supplementary_materials_legends_revised.pdf]

Supplementary information for

Three-Dimensional Visualization and Detection of the Pulmonary Venous-Left Atrium Connection Using Artificial Intelligence in Fetal Cardiac Ultrasound Screening

Reina Komatsu, Masaaki Komatsu, Katsuji Takeda, Naoaki Harada, Naoki Teraya, Shohei Wakisaka, Takashi Natsume, Tomonori Taniguchi, Rina Aoyama, Mayumi Kaneko, Kazuki Iwamoto, Ryu Matsuoka, Akihiko Sekizawa and Ryuji Hamamoto.

The file contains
The legends of Supplementary Figures S1-S3, Supplementary Table S1, and Supplementary Video S1

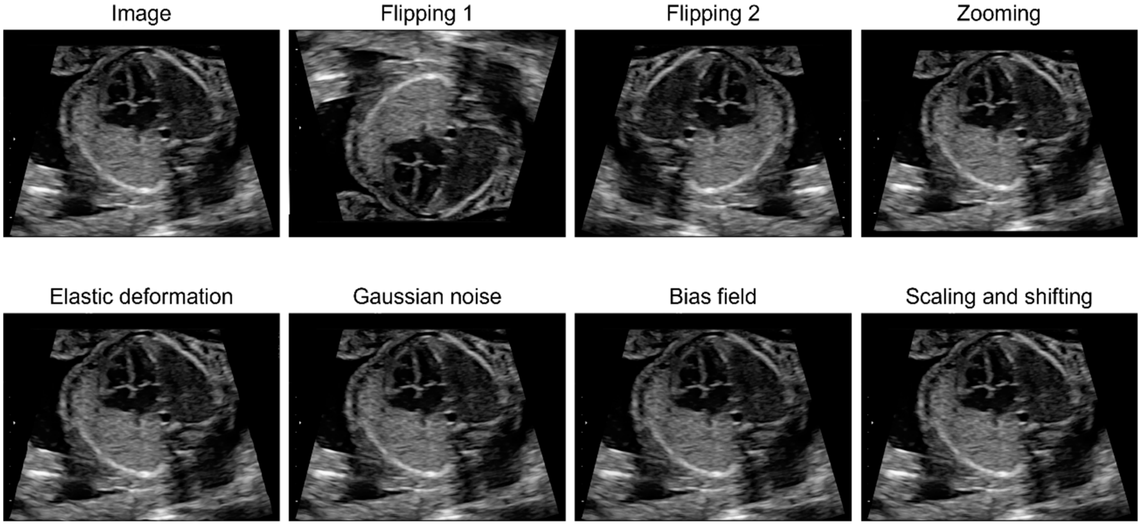

Supplementary Figure S1. Representative augmented images after data augmentation.

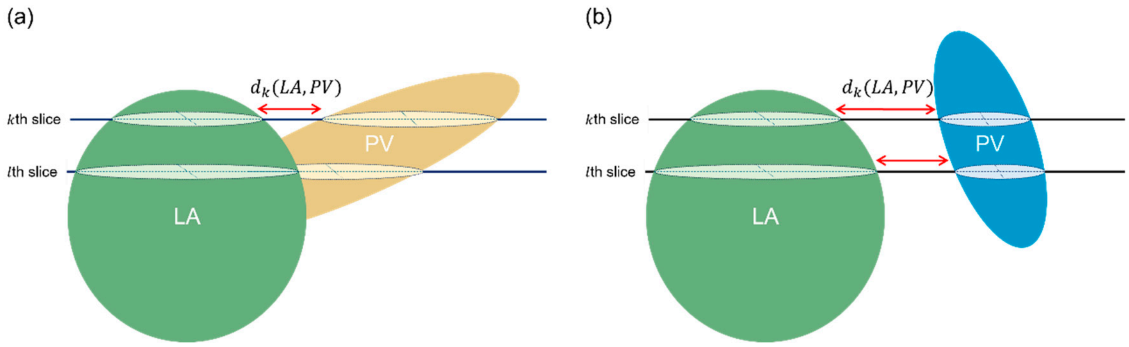

Supplementary Figure S2. Schema of PV-LA connections for the PLD calculation of a normal case (**a**) and a TAPVC case (**b**). $d_k(LA, PV)$ represents the shortest distance between the PV and LA in each slice. PV, the pulmonary venous; LA, the left atrium.

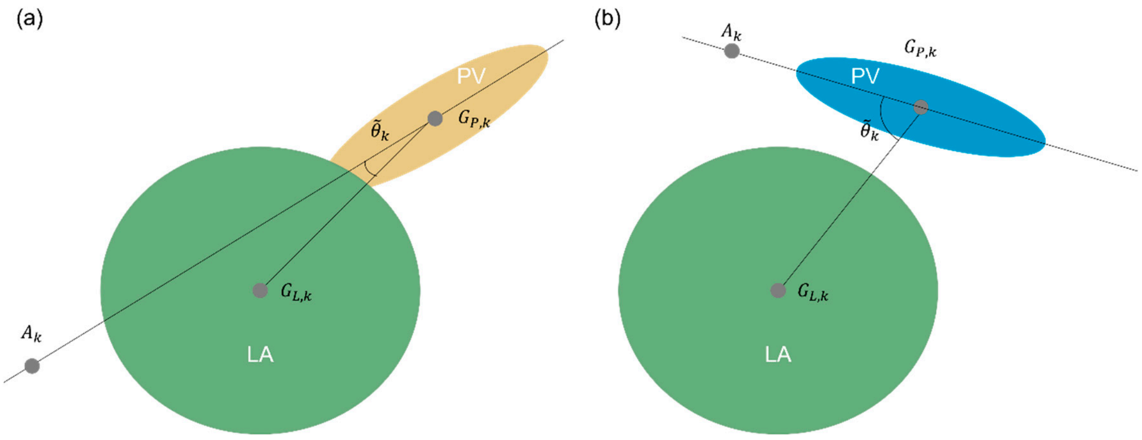

Supplementary Figure S3. Schema of PV-LA connections for the PLA calculation of a normal case (**a**) and a TAPVC case (**b**). $\tilde{\theta}_k$ represents the major axis angle between the PV and LA in each slice. PV, the pulmonary venous; LA, the left atrium.

Supplementary Table S1. The default hyperparameters for each model.

Supplementary Video S1. Fetal cardiac ultrasound screening. Each video comprised sequential cross-sections from the level of the stomach through the heart to the vascular arches, mainly in the apical view. The pulmonary veins are indicated by yellow arrows.
