# Supplementary figures and images for: Three-Dimensional Visualization and Detection of the Pulmonary Venous–Left Atrium Connection Using Artificial Intelligence in Fetal Cardiac Ultrasound Screening
Source: Bioengineering (Basel). 2026 Jan 15;13(1):100. doi: 10.3390/bioengineering13010100 (PMC12837485; doi:10.3390/bioengineering13010100)

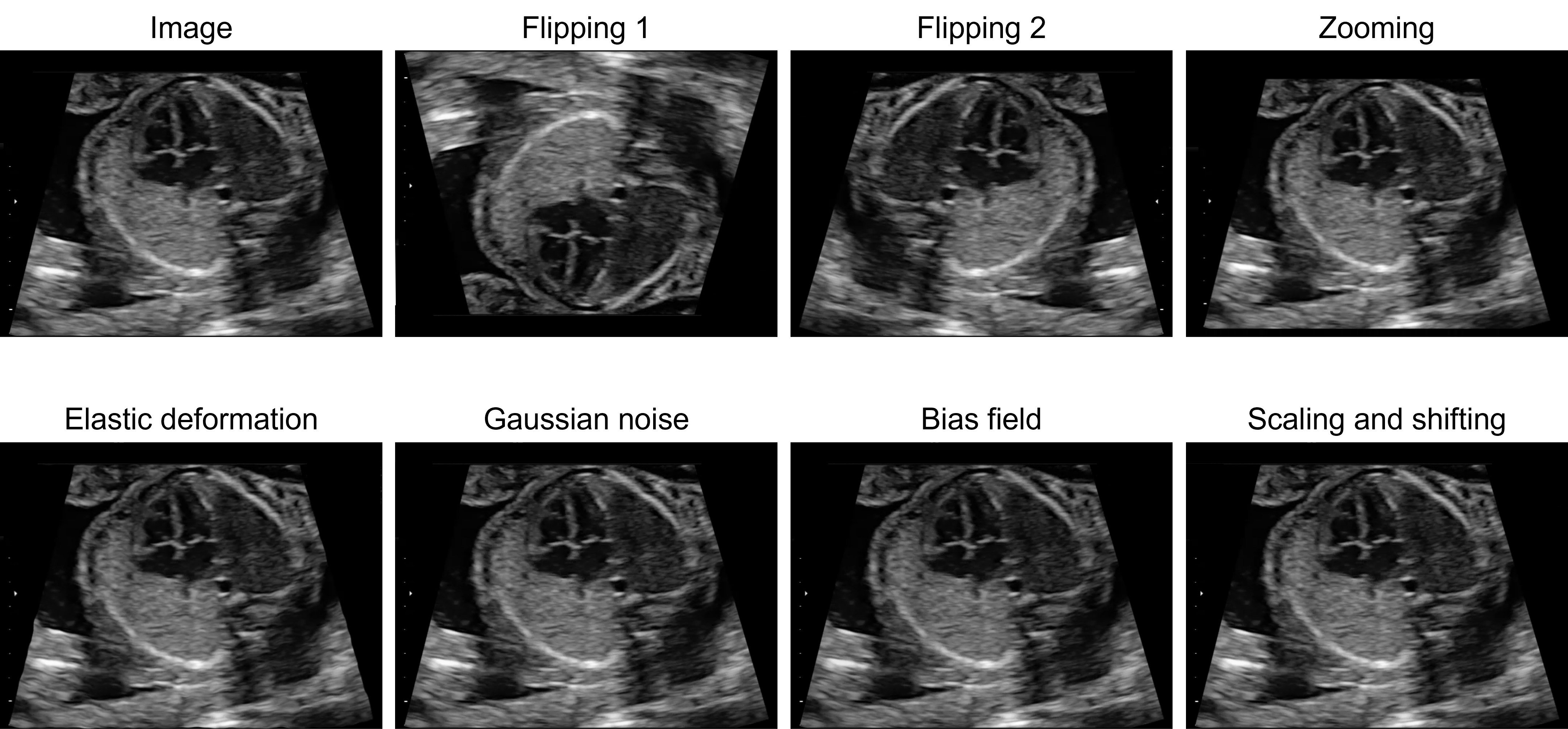

Supplement: Supplementary file 1 [file bioengineering-13-00100-s001.zip › Figure S1.png]

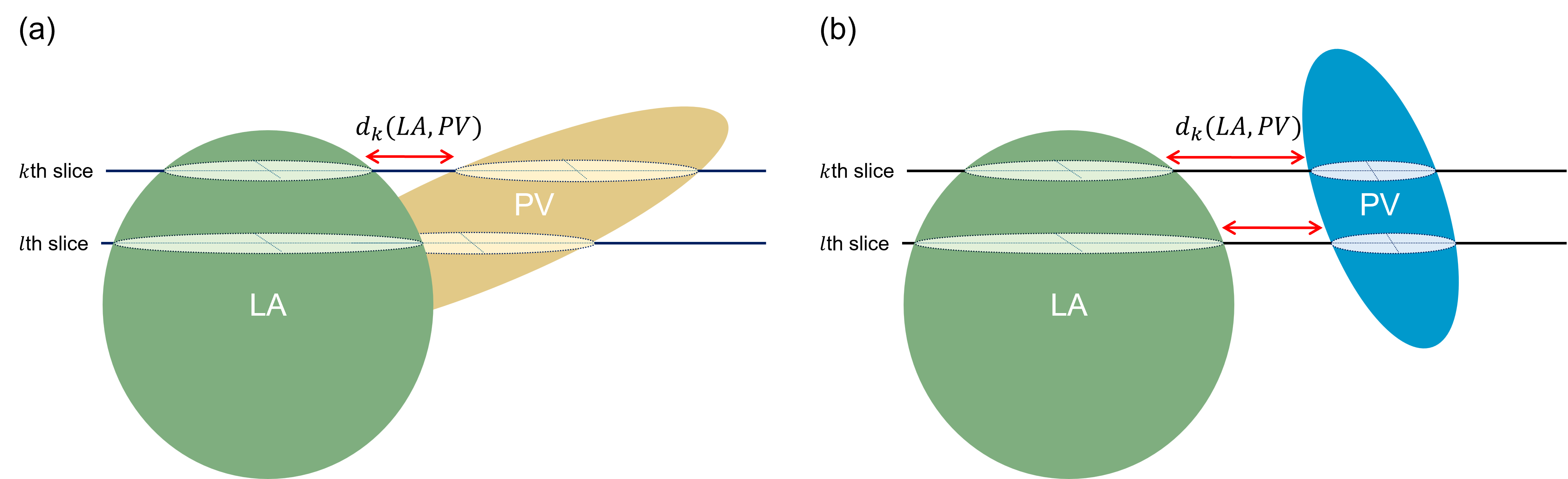

Supplement: Supplementary file 1 [file bioengineering-13-00100-s001.zip › Figure S2.png]

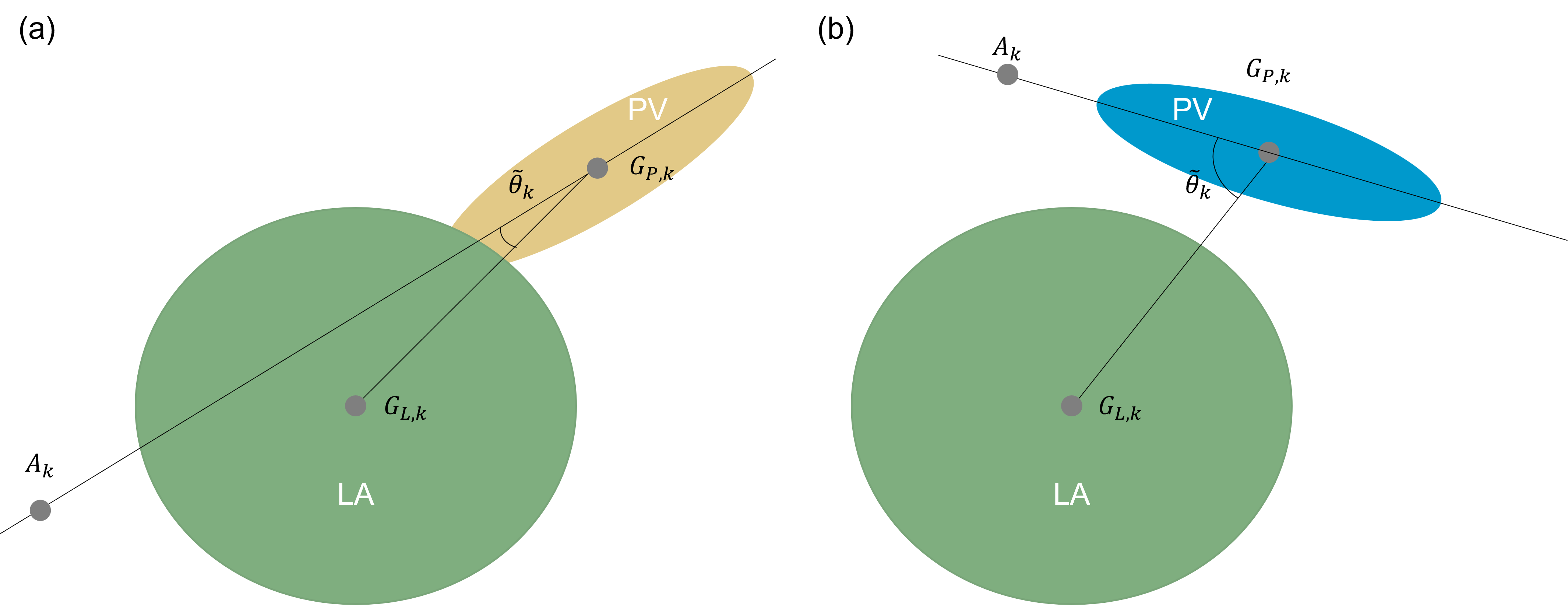

Supplement: Supplementary file 1 [file bioengineering-13-00100-s001.zip › Figure S3.png]
